# A guide to a new short course to promote interest and engagement in psychiatry in medical students

**DOI:** 10.1192/pb.bp.114.046797

**Published:** 2015-08

**Authors:** Matthew Langley, Benjamin Lomas, Zena Schofield, Gillian Doody

**Affiliations:** 1 East Midlands North School of Psychiatry; 2 University of Nottingham

## Abstract

This article describes a new course for preclinical medical undergraduates designed to promote interest and engagement in psychiatry. The course employed a range of innovative teaching techniques alongside ward visits to provide students with early clinical experience. Unusually, assessment for the course involved the production of creative works as well as reflective writing about students' experiences. We collected a variety of feedback from participants showing that they found the course enjoyable and educational. We conclude that, overall, the course had a positive effect on student perceptions of psychiatry.

Psychiatry is one of several medical specialties undergoing a crisis in recruitment, with a 15% shortfall in core trainee (CT1) allocations year by year.^1^ The Royal College of Psychiatrists is seeking to address this deficit by engaging with students before, during and after medical school,^2^ with recommendations for interventions at each level. One such intervention is the provision of student selected components (SSCs) in psychiatry. In this paper, we discuss our experience of developing and delivering an innovative optional module for second-year medical students studying for the Bachelor of Medical Sciences (BMedSci) degree at the University of Nottingham and the impact this had on their attitudes towards psychiatry.

## Aims and objectives

The aims of the module were:

- to stimulate students to learn more about psychiatry before entering traditional 6-week clinical placements in year 4
- to demystify and destigmatise the specialty and consider how psychiatric practice differs relative to the traditional medical models of medicine and surgery
- to improve students' critical reflection skills and to allow them to improve their medical practice by reflecting on personal experience.

When designing this course, we were guided by our aims and objectives and by literature available about other psychiatry SSCs (see below). We were, however, constrained by some practical considerations. The module had to fit into an existing undergraduate timetable that was already very busy. This meant that the SSC had to be completed in one semester and we managed to negotiate eight sessions of varying length for our teaching. The total duration of the course, including time for submitting the assessments, was approximately 3 months. We were also keen to avoid duplicating the teaching and experiences that students would receive during their clinical attachments to psychiatry in their fourth year.

A literature search was conducted to see whether other medical schools had undertaken similar SSCs and, if so, what their experiences were. A range of databases (AMED, Embase, HMIC, MEDLINE, PsycINFO, BNI, CINAHL, Health Business Elite) were searched using all combinations of the terms ‘psychiatry’, ‘student selected component’, ‘special study module’, ‘SSC’ and ‘SSM’. This search yielded 21 results and, after removal of duplicates, review of the abstracts identified 4 articles of particular interest. Brown *et al*'s^3^ survey of Scottish psychiatrists supported our view that engaging medical students early in their training was important for promoting positive attitudes towards psychiatry. We also found three published examples of SSCs/SSMs in psychiatry. One of these was a 3-week SSC at Hull York Medical School on mental health and the arts.^4^ The other two were both SSMs looking at the representation of mental illness in films.^5,6^

Some parts of our design were influenced by these reports. For example, we included short examples from films and other popular media in our seminars (e.g. *Winnie the Pooh* cartoons). We also acknowledged the natural link between mental health and the arts in our innovative assessment scheme where students were asked to prepare and present creative pieces influenced by their experiences during the module. However, we felt that a design using only one approach (e.g. film or arts in general) would not allow us to meet all of our objectives. Hence, our course included a variety of teaching modalities and, very importantly, used early clinical experience in the form of structured ward visits.

The module structure comprises lectures, small-group teaching, student-led presentations, group work, self-directed learning and two field trips to psychiatric hospitals. The intention was not necessarily to recruit every member of this group to the specialty, but to sow the seeds of positive regard for psychiatry and its patients and to destigmatise mental illness.

The course consists of five 2-h seminars on the following topics: the history of psychiatry, psychosis, neurosis, the subspecialties and ethical dilemmas in psychiatry. Interspersed with these seminars are two clinical visits supervised by senior clinicians, each 3 h long. Twelve students are accommodated on the course. This early exposure to mental health in-patient care is to our knowledge rare in the UK. In the final 3-h seminar students present part of the course assessment in the form of a creative piece about psychiatry to an audience of their peers, consultants and an assessment panel.

Interesting and interactive sessions were devised which minimised didactic teaching, as far as possible, while still introducing the serious and sensitive nature of mental illness. It was also important not to duplicate any of the curriculum material the students would receive in their later fourth-year clinical psychiatry placements.

## Course structure and materials

### Seminar 1: History of psychiatry

An assumption was made that students' prior knowledge was comparable to a member of the public's and therefore likely to be influenced by popular media portrayals of mental illness^7^ or personal experience involving themselves, friends or relatives.

The session begins with students having to define the job descriptions of psychologists and psychiatrists in an interactive session with the facilitators for 20 min.

‘Getting away with murder’ is then played. This is an exercise designed to draw out student preconceptions and prejudices, which could then be challenged and revisited throughout the course. Students are divided into two groups and asked about the role mental illness plays in violence. A scenario is presented whereby students are asked to imagine they have committed a murder and have decided to feign symptoms of a mental illness to be found not culpable. Working in their groups facilitated by course tutors, the students then brief a patient simulator (who is experienced in portraying the symptoms of mental illness in medical assessment and training) on how to behave and what to say. Next, a specialist forensic psychiatry trainee interviews the simulators in front of both groups. Students are positively rewarded with chocolate for the simulator portrayal of a convincing symptom or syndrome of mental disorder.

This exercise successfully sparks the students' interest and leads to an open discussion, guided by feedback from the interviewing psychiatrist, about the symptoms the students describe and why they believe them to represent mental illness. The symptoms generated by the students were contrasted with symptoms that are typically seen in forensic psychiatry settings. The remainder of the session is spent presenting students with a broad history of psychiatry through the ages with a focus on attitudes towards mental illness in different societies and contrasting these with the students' own attitudes.

### Seminar 2: Psychoses

The second seminar focuses on the nature of psychosis and the challenges it poses to patients, mental health services and society. The students are shown a video interview with a patient who experiences features of psychosis. To facilitate empathy, the patient selected for interview was also a student of a similar age. He talks through his experiences – the first time the students hear a description of a psychotic episode – and the impact the illness has had on his life. This challenges the students' previously expressed views as to the experience of mental illness and the information generated by students during the ‘getting away with murder’ game is revisited. A discussion is facilitated to establish the nature of the symptoms of psychosis.

To enable students to develop an experiential insight into schizophrenia, we attempted to simulate a psychotic symptom. There is an existing body of literature to support this approach.^8^ The experience of an auditory hallucination is simulated for the students by listening to an audio file played on their own mobile phones or other devices via headphones. The scripts were based on patients' descriptions of their auditory hallucinations and then anonymised. The hallucinations were voiced by members of the teaching team, recorded and mixed together. The audio file was distributed to students via the Moodle virtual learning environment (https://moodle.org) with instructions to bring it to the relevant session on a device with headphones attached, having not yet listened to it.

The pressure to perform well academically in medical students is well known, as is their competitive nature. A quick-to-administer intelligence test was identified and students take the test under normal conditions. Next, they complete a comparable test while listening to the simulated auditory hallucinations on their headphones. The tests are scored and students compare their two sets of individual results; group means are calculated and a paired *t*-test performed to demonstrate the effects of hallucinations on intelligence testing.

### Seminar 3: Neuroses

This seminar aims to introduce depression and anxiety disorders by highlighting issues related to defining the boundaries between normal and pathological experiences. A small degree of anxiety is generated in students at the beginning of the session. On arrival they are told they are to sit an *ad hoc* examination under strict conditions. The teaching team act in an anxious manner themselves, talking about the need to standardise the course for external examiners, while students wait silently for everyone to arrive. When instructed to turn the exam paper over students discover a questionnaire asking about the acute symptoms of anxiety derived from the ICD-10 criteria for generalised anxiety disorder. Students then reflect on their feelings on being told they had an examination and how these match to those probed in the questionnaire. A number of anxiety symptoms are elicited. Having induced symptoms of anxiety, prior learning is then activated by a group discussion reminding the students of the biological basis of the physiological changes that occur in anxiety.

To further illustrate the concepts included in the broad area of neurosis, video clips of characters from *Winnie the Pooh*^9^ are then shown and discussed in the group. This illustrates how disorders can be identified (e.g. depressive disorder in Eeyore, anxiety disorder in Piglet) and the importance of having a structured means of drawing the line between the normal and the pathological.

It was considered important that issues of suicide and risk assessment are discussed. To facilitate this, examples of famous people who have died by suicide are called upon, allowing this emotive topic to be discussed in a sensitive way within the now-bonded group.

### Seminar 4: Subspecialties

As psychiatry is a medical specialty with many subspecialties that most medical students do not get experience in, the students were introduced to the main subspecialties with brief talks from consultant psychiatrists in various fields (old age, child and adolescent, intellectual disability, forensic and psychotherapy). Exposure to the enthusiasm and expertise of specialist consultants is a valuable part of the module and speakers are briefed to talk to the topic ‘I like my job, because … ’ for 20 min and allow 5 min for questions. Following this seminar, students are asked to express individual preferences for their visit to a subspecialty unit.

### Seminar 5: Ethical dilemmas in psychiatry

Psychiatrists face numerous clinical ethical dilemmas. It is important to introduce the students to these issues; they are taught ethical principles at an early stage in the medical curriculum and have a basic understanding of the area. Consultants from subspecialist areas within general adult psychiatry present to the students real clinical cases featuring ethical considerations. The consultants specialise in liaison psychiatry, perinatal psychiatry, eating disorders and gender identity issues. Feedback from students indicated that, before the presentations, they were unaware of some of these specialist areas of psychiatry.

### Clinical visits

Within the module are two visits to in-patient psychiatric units. Students attend in pairs: visiting one of the six local acute general adult wards on the first visit and one of the available subspecialty in-patient units on the second visit (drug and alcohol, perinatal, forensic, intellectual disability, child and adolescent, old age). To prepare the students for the visit, information is given about ward etiquette (dress, ID, behaviour, safety), suitable questions are suggested for the meeting with a patient and opportunities are given to ask questions about the visits. Senior doctors, either consultants or higher specialist trainees, lead the visits. The visit format is prescribed as follows. First, students observe the psychiatrist interviewing a consenting patient (30 min) and then discuss the case. After a coffee break, students are introduced to a consenting patient to speak with them in a communal ward area for 30 min. Following this, students have the chance to reflect verbally on their experiences with the psychiatrist and ask questions about the patient they have seen. By encouraging the students to focus on the impact of the mental illness and the care received, rather than take a formal psychiatric history, the importance of and need for high-quality psychiatric care is reinforced.

### Assessment

As an optional module contributing towards an intercalated BMedSci degree, a summative assessment is mandated. As the aim of the module is to demystify and destigmatise psychiatry as well as developing empathy the assessment places emphasis on reflective processes. Students are required to produce two reflective essays, one entitled ‘My impressions of psychiatry’ (1500 words) and the other ‘Meeting a psychiatric patient’ (1000 words). They are also required to produce a piece of creative work that communicates their understanding of any aspect of psychiatry with an accompanying written explanation of the work. This was presented to the student group, the facilitators, consultants and psychiatric staff who facilitate the ward visits. A psychiatric occupational therapist helped to develop the marking criteria and was a member of the assessment panel. The creative work presented was emotionally poignant and exceeded expectations; students produced paintings, photography, poetry, interpretative contemporary dance, short films and sculptures. Their work has been exhibited at the medical school and is available for future groups to see online.

## The student experience

The Attitudes to Psychiatry (ATP) questionnaire^10^ and a bespoke questionnaire to obtain qualitative and quantitative feedback on the creative assessment and the role of reflection in medical training were completed by students both before and after the course. There were no statistically significant differences in attitudes to psychiatry question items before and after the course. There were 11 students who completed the ATP before the module and 10 who completed it at the end of the module. When individual statements were examined using chi-squared results for the responses before and after the module compared with the mean response before the module for each question on the ATP, three questions initially seemed statistically significant (Question 3: ‘Psychiatric hospitals are little more than prisons’, Question 9: ‘Psychiatric teaching increases our understanding of medical and surgical patients’, Question 29: ‘Psychiatric patients are often more interesting to work with than other patients’). However, once adjusted for multiple testing using a Bonferroni correction, the results were no longer statistically significant. The lack of statistically significant results in the ATP is probably due to the small sample size and the fact that students self-selected the course, which might have resulted in the group being biased positively towards those attracted to psychiatry at the outset.

The most positive effect of the course was apparent in the essays the students produced. Most chose to reflect on their personal journey through the course, with a common theme being the realisation that previously held stereotypic views of the psychiatrist and their patients were inaccurate. Our aim to give students a positive experience of psychiatry was achieved – one student wrote ‘Considering that I chose this module having no intention of taking a career route in psychiatry, I must say that the module has certainly left positive impressions upon me, and right now, I definitely would not rule out such a possibility’.

An example of the impact of the module is voiced by a student who wrote: ‘My impression of psychiatry has been very positive. [Psychiatry] is an exciting and ever-evolving profession’. Admittedly, students were aware of the ‘hidden agenda’ created by the recruitment crisis in psychiatry and perhaps they knew we would be delighted to read quotes such as, ‘I have ended this course wanting to pursue a career in old age psychiatry’.

Another common essay theme was the belief that medical students should be taught about psychiatry earlier in their training. One student stated: ‘My impressions of psychiatry have changed dramatically over the [past] year and I am of the opinion that all medical students would benefit from early exposure to this [specialty]’. Another student, having highlighted the stigma among medical students towards people with mental illness, said: ‘I believe there should be a lot more emphasis on mental health in … the preclinical phase of medical training, perhaps this can reduce the stigma associated with the subject’.

Clinical visits received excellent feedback from the students. One wrote: ‘Probably the most important factor in sculpting my impressions, were my own experiences on the wards … It felt like no other hospital ward that I'd been on before’. Another student wrote: ‘Experiences such as meeting this patient, and the ward round, changed my views and attitudes towards psychiatry entirely’. The effect of the contact with patients was clear, with one student writing: ‘Thinking about psychiatry now, the patients are what I will take from this module’.

The aim to reduce stigma both towards psychiatric patients and their psychiatrists appears to have been achieved. One student stated ‘I was greeted, not with a room of [Freuds], but kind, friendly, relaxed psychiatrists. People who seemed like the most passionate doctors of any [specialty] I'd encountered’. Another said: ‘[The course] has stripped away levels of stigma I barely knew I had, yet which society had conditioned me into having’. Several of the students commented on their desire to see stigma towards those experiencing mental illnesses to be reduced, typified by one student who wrote ‘Mental illness is just as real as physical illness and deserves the same amount of respect’.

## Discussion

It should be noted that the sample size of the first cohort was small, only 12, so it is difficult to draw anything more than impressions from the data collected. The course has been repeated but again with only 12 participants. Because funding streams are different for preclinical and clinical medical students at present there are practical limitations on the number of students who can undertake the module. However, with minor alterations, the module could be taught with larger numbers of students and in medical schools throughout the UK. A useful follow-up would be to conduct the ATP on second-year students who do not undertake this module and compare the results. In addition, following up this group of students to see whether the changes in attitude are sustained or short-lived might be of value. Comments made by the students during the module indicated that negative attitudes regarding mental health services are being propagated even in preclinical teaching sessions. As it has not been possible to do a long-term follow-up of these students yet, the effect of studying other areas of clinical medicine on their long-term career choice has not been established. This positive change in attitudes to psychiatry may or may not be sustained in the long term, but at least we have a tool that has demonstrated a positive impact on medical students that could be repeated in larger numbers.

## Summary

In conclusion, by using innovative, interactive teaching techniques combined with early clinical visits to psychiatric units, the course achieved its aims of demystifying and destigmatising psychiatry. At the start of the course, none of the students had considered psychiatry as a career; by the end 25% stated they intended to become psychiatrists and a further 17% said they would consider a career in psychiatry. More than half of the students requested to do their BMedSci projects in areas related to psychiatry through the Institute of Mental Health, Nottingham. The remainder acknowledged a positive change in their attitudes towards psychiatry, viewing it as an interesting and important medical specialty. We are currently exploring the possibility of offering a similar bespoke course for local sixth-form pupils to encourage those interested in psychiatry to apply to medical school.

The assessment work demonstrated that all the students had increased their knowledge of psychiatry, with most having undertaken further personal study that was referenced in their submissions. The assessment process included a reflective piece enabling the students to develop reflective skills, with some being more able to demonstrate this in written form than others. As students are motivated by assessment, using an assessed reflective piece encouraged their focus on this skill that will benefit them in their future medical careers. Psychiatry in particular is an area where reflective practice is vital, so this assessment tool has face validity. We would encourage all psychiatrists in medical education to reflect on how we can make changes to improve recruitment of appropriately skilled doctors to psychiatry and present this module as one suggestion.
